# Strategies to facilitate effective caring for patients in primary health care clinics

**DOI:** 10.4102/curationis.v44i1.2201

**Published:** 2021-11-30

**Authors:** Tinswalo Nesengani, Charlene Downing, Marie Poggenpoel, Chris Stein

**Affiliations:** 1 Department of Nursing, Faculty of Health Sciences, Nelson Mandela University, Gqeberha, South Africa; 2 Department of Nursing, Faculty of Health Sciences, University of Johannesburg, Johannesburg, South Africa; 3 Department of Emergency Medicine, Faculty of Health Sciences, University of Johannesburg, Johannesburg, South Africa

**Keywords:** strategies, professional nurse, facilitating, effective caring, primary health care clinics, patients

## Abstract

**Background:**

Caring is described as the innermost core of nursing which occurs in a relationship between the patient and the care provider. Although caring in nursing is associated with maintaining and strengthening of the patient’s sense of dignity and being a person, there seems to be a gap between caring theories in nursing, healthcare policies and caring for patients by professional nurses in primary health care clinics. Developing strategies that will facilitate effective caring for patients by professional nurses in primary health care clinics within an ethical and mindful manner became an area of focus in this study.

**Objectives:**

To develop strategies to facilitate effective caring for patients by professional nurses in primary health care clinics in South Africa.

**Method:**

Strategies were developed based on the conceptual framework developed in Phase 2, which was derived from synthesis of the results of Phase 1 of the previously conducted study and supported by literature. The conceptual framework reflects the survey list of Dickoff, James and Wiedenbach’s practice theory.

**Results:**

Three strategies were developed: 1) facilitating maintaining of the empowering experiences; 2) facilitating addressing the disempowering experiences by professional nurses, and 3) facilitating addressing of the disempowering primary health care clinic systems.

**Conclusion:**

The developed strategies, being the proposed actions, procedures and behaviours, could facilitate effective caring for patients by professional nurses in primary health care clinics.

## Introduction

Caring Science is based on the philosophy of Human Caring, a theory articulated by Watson as a foundational covenant to guide nursing as a discipline and profession (Durant et al. 2015:e136). Caring is described as a value and an attitude that manifests itself in the form of a concrete act (Andersson et al. 2015:1; Rhodes, Morris & Lazenby 2011:5). Nikfarid et al. (2018:2) indicate that Watson’s Human Caring Theory places importance on the concept of caring as the essence of nursing. In nursing, caring becomes the basis for carrying out the task and the willingness to do it, therefore, considered principle and ethics-based (Armstrong 2013:148; Pai, Eng & Ko 2013:424). Based on the above-mentioned facts, caring must be present if nursing is to be truly effective and to give patients a feeling of respect. Although caring in nursing is associated with maintaining and strengthening of the patient’s sense of dignity and being a person (Andersson et al. 2015:1), there seems to be a gap between caring theories in nursing, healthcare policies and the clinical practice in primary health care (PHC) clinics.

## Background

In South Africa, the implementation of the PHC approach through the District Health System (DHS) is considered as a fundamental component of the health transformation process in post-apartheid South Africa. The PHC approach’s strength is based on its response to the local needs of individuals, families and populations through a comprehensive, intersectoral approach that focuses on communities as the unit of intervention (Dookie & Singh 2012:2). According to Mayers (2010:3), the implementation of the PHC approach as a policy by the National Department of Health (NDoH) in South Africa since 1994 indicates a significant paradigm shift in the provision of health care services. Mofolo, Heunis and Kigozi (2019:2) mention the fact that based on the NDoH’s policy and commitment on building and providing of PHC services in South Africa’s health care system, the focus is on making PHC clinics as the point of entry for patients in need of preventative care, diagnosis and treatment of minor ailments. Mayers (2010:4) indicates that according to the South African PHC approach, nurses are often found to be the first contact of care, the only connection to health care whilst at the same time being considered as the major health care providers.

Some countries in the African continent, including Zimbabwe (Ray & Masuka 2017:1), adopted the PHC approach two years after the Alma Ata Declaration in 1978. The PHC approach was implemented by directing the health resources towards the disadvantaged areas by active participation of communities in transforming their health. Primary health care services in Zimbabwe are largely nurse-led, with PHC nurses based in rural areas. Like in South Africa, the nurses’ caring activities concentrate mainly on preventive, promotive, curative and rehabilitative healthcare provision for individuals, groups and communities. Namibia is another country which adopted the PHC approach and made a commitment to health as a fundamental human right, in response to gaining independence from South Africa in 1990. The country’s focus was on reforms to transition on a health care system based on a central role for PHC. The PHC services are rendered by nurses, whose caring activities include referring of patients to health centres when the patients’ needs exceed their scope of practice or available resources (Christians 2020:1).

Henderson et al. (2014:337) indicate that in common with many countries, Australia expanded the role of PHC to manage the growing burden of chronic diseases and to prevent hospitalisation, which associates PHC with practice nurses. Unlike in South Africa where it is required of professional nurses working in PHC clinics to possess an additional qualification in PHC nursing, practice nurses in Australia can be enrolled or registered who are employed to provide services for general practice. Their caring responsibilities include undertaking health assessments and health prevention activities: history taking, clinical examinations, organising investigations, implementing a management plan or preventative health care (Henderson et al. 2014:338).

On the other hand, even though the PHC approach was envisaged as ‘the first level of contact’ of individuals, families and communities, and to bring health care as close as possible to where people live and work, the vision remains unfulfilled and overlooked in the developed parts of the world (Rao & Pilot 2014:1). The United States serves as an example that provides a striking imbalance between PHC and specialist care, which is driven by the availability of specialist diagnostic and therapeutic options based on advanced technologies and insurance-based health financing (Rao & Pilot 2014:2).

Although Dookie and Singh (2012:2) indicate that PHC is implemented as a public health strategy derived from the social model of health care and based on the philosophy that health gains are better obtained when people’s basic needs are met first, PHC services in South Africa still face challenges in responding adequately to the local needs of individuals, families and populations. A research study conducted by Mayers (2010), explored the experiences of nurses in PHC of South Africa. The findings revealed that there were challenges in the provision of PHC in the public sector, which included long waiting times, excessive workloads for staff, poor attitudes, rudeness and favouritism of nurses, accompanied by lack of confidentiality – particularly in large urban clinics. Other factors such as an inadequate distribution of resources, inadequate or irregular supply of basic medicines and the inefficient interactions amongst different hierarchical levels of public health services were also mentioned, which were considered as factors that further compromised the services provided. Mayers (2010:4) further highlights that even though according to the South African PHC approach, nurses are found to be the first contact of care; it has also been acknowledged that these nurses find themselves increasingly responsible for a service that is under-resourced in terms of human and material resources whilst being referred to as the backbone of the health care system. In many ways, as confronted with challenges, these nurses are seen to fill up gaps in a system which is lacking in the resources which are needed to address the many healthcare related needs (Mayers 2010:4).

Results from a research study, ‘The nature of community health care centre practice environments in a province in South Africa’, confirm that the state PHC services in South Africa offer essential first contact health care, but nurses in PHC clinics face many challenges in their practice environments (Rabie, Coetzee & Klopper 2016:29). Such challenges are said to include nursing shortages, lack of resources and inadequate support, lack of leadership, accompanied by higher levels of job dissatisfaction. The reasons for job dissatisfaction were cited as high workloads, poor wages, lack of career development opportunities, resulting in a hampered public health care in an already resource-deprived system. Despite the various dis-enablers having been reported in the delivery of health care services in the public health PHC system, little is known about the strategies developed to facilitate effective caring for patients by professional nurses in PHC clinical settings.

Immense work has been performed by other healthcare professions in developing strategies to deal with the challenges they face whilst caring for patients. Various strategies developed include, for example Shaikh and Roth (2017). Strategy 1 focused on understanding the population served and generating actionable information. Activities included identifying and closely coordinating care super-utilisers, which was to help derive clinical decision making at the point of care. Super-utilisers are regarded as those individuals seen to consume a relatively large share of the health care resources. Strategy 2 focused on redesigning PHC through building of new partnerships, team-based care and group visits, as well as utilisation of telematics and digital media. Strategy 3 focused on prioritising health promotion through integration of interventions that address social determinants. Strategy 4 was codesigning and coproducing care with patients, done through patients’ experiences and in-patient meetings with representatives to discuss their recent experiences in healthcare. Strategy 5 focused on engagement and empowerment of the frontline staff and learners in care transformation by engaging staff, clinicians, students and trainees in quality improvement initiatives.

*Toward a unified Integration Approach: Uniting Diverse Primary Care Strategies under the Primary Care Behavioural Health (PCBH) Model* (Sandoval et al. 2018), focused on developing a model of service delivery which included patient-centred care as well as strategies that could improve clinical outcomes, costs and primary care provider satisfaction. The focus for *Developing support strategies for burn care nurses through an understanding of their experiences: A meta-ethnographic study* (Bayuo et al. 2019) was on the development of supportive nursing teams as the burn care environment was noted to present numerous stressors. This was in response to nurses observed to use coping strategies such as creating a barrier, suppressing or masking emotions and distancing.

In Alm-Pfrunder et al. (2018), nurses used a palette of strategies for communication whilst assessing patients’ needs. The use of all their senses was also described as an additional strategy used by nurses, and an important part of the assessment. Two workshops were conducted by Cummins et al. (2018), with the aim being to introduce students to sensitive topics and to promote self-care strategies to support students in their transition to become midwifery students. Lastly, ‘With an open heart we receive children’: *Caregivers’ strategies for reaching and caring for street children in Kenya* (Kaime-Atterhӧg, Persson & Ahlberg 2017) describe strategies which included collaboration by caregivers with other stakeholders such as governmental correctional centres for children who are in conflict with the law, to deal with difficult cases, provision of a variety of recreational activities, health care and counselling, as well as offering children vocational training within and outside the home.

Dookie and Singh (2012:1) reveal that despite South Africa’s progress and attempts at implementing PHC, various factors are still noted to limit its success, which corroborate with the findings in Nesengani (2019:68). Based on the PHC approach, Dookie and Singh (2012:2) indicate that patients should have access to affordable and appropriate healthcare services. To ensure upholding of caring as a therapeutic intervention and maintaining PHC principles such as efficiency and effectiveness in health care service delivery it became the factors that motivated the researchers to develop strategies to facilitate effective caring for patients by professional nurses in PHC clinics. Therefore, the focus of this article is on developing strategies to facilitate effective caring for patients by professional nurses in public PHC clinics of South Africa.

## Research design and method

This study was part of a larger doctoral study that was conducted in four phases. In Phase 1, the experiences of caring for patients by professional nurses in PHC clinics were explored and described. A qualitative, exploratory, descriptive and contextual research design (Brown 2014:37; Christensen, Burke Johnson & Turner 2015:68; Holloway & Wheeler 2010:93; Houser 2015:135; Moule & Goodman 2014:173; Patton 2015:125) was utilised. In Phase 2, a conceptual framework was developed, based on the results from Phase 1. Strategies to facilitate effective caring for patients by professional nurses in PHC clinics were developed in Phase 3, based on the conceptual framework developed in Phase 2. This article will only report on developing of strategies to facilitate effective caring for patients by professional nurses in PHC clinics.

The conceptual framework was developed from the results gained in Phase 1 of the study. The themes derived from the findings were as follows: 1) Empowering experiences in caring for patients, 2) Disempowering experiences in caring for patients and 3) Disempowering experiences with PHC clinic systems. Literature regarding: 1) facilitating maintaining of the empowering experiences, 2) facilitating addressing the disempowering experiences, 3) facilitating addressing of the disempowering PHC clinic systems were the guiding principles in developing the strategies. The strategies in this study entail proposed actions to be taken, behaviours to be adopted and procedures to be performed to facilitate effective caring for patients by professional nurses. The strategies also include a cluster of decisions to be taken to pursue the goal to facilitate effective caring for patients by professional nurses. The conceptual framework was developed from the researcher’s thinking map as the basis for developing strategies, reflecting the practice theory survey list of Dickoff, James and Wiedenbach (1968:415–435). The process involved identifying and defining concepts, as well as proposing relationships between those concepts. The six main concepts of the Dickoff et al. (1968)’s practice theory survey list, namely *agent, recipient, context, procedure, dynamics and outcome* provided the principles that underpin nursing practice and helped to generate further nursing knowledge and new ideas.

The conceptual framework in this study reflects the activities or the strategies undertaken by the nurse manager and professional nurses to facilitate effective caring for patients by professional nurses in PHC clinics. The objectives to be achieved, proposed actions to be taken and how the available resources will be used to achieve the goal of effective caring for patients by professional nurses were listed. The decisions regarding the procedures to be implemented and the behaviours to be adopted were also made. The conceptual framework enabled the researchers to interpret and link the study findings into practice and also served as a tool for connecting concepts to provide a context for interpreting the study findings. The questions of the survey list were as follows: Who is the agent?, Who is the recipient?, What is the context, What is the procedure or process?, What are the dynamics?, and What is the purpose or outcome?

A brief outline of the six elements follows:

- Agent – Who performed the activity?
  The nurse manager is the agent who plays an active role in facilitating the process of professional nurses effectively caring for patients. The facilitative role focuses on maintaining empowering experiences and addressing disempowering experiences through a continuous interactive process that ensures engagement, support and empowers professional nurses by guiding them in realising the nursing goals. The nurse manager also plays a significant role in facilitating the implementation of activities that promote the delivery of effective caring for patients by professional nurses in accordance with the patients’ health needs.
- Recipient – Who was the recipient of the activity?
  The professional nurses are the recipients who receive guidance and support from the nurse manager on maintaining empowering experiences and addressing disempowering experiences whilst they actively engage themselves and play an active role in the facilitation and implementation of the process to facilitate effective caring for patients.
- Context – In what context was the activity performed?
  The context for this study is the PHC clinics rendering comprehensive PHC services to patients; the nurse manager and professional nurses caring for patients in PHC clinics. The nurse manager supports and often takes part in caring for patients, especially when the PHC clinics are too full, to off-load the heavy workload from the professional nurse.
- Procedure – What was the guiding procedure?
  The outline of the procedure in this study is to develop strategies to facilitate effective caring for patients by professional nurses in PHC clinics. The nurse manager, being the agent, performs the activities of the facilitation procedure in interaction with professional nurses. The professional nurses, are the recipients of the activities of the facilitation procedure, aimed towards the realisation of the outcome of the study.
- Dynamics – What was the energy source for the activity?
  The dynamics are the driving forces or motivation to provide effective caring for patients. The empowering and disempowering experiences encountered by the professional nurses and the patients in the PHC clinics motivate the nurse manager in facilitating effective caring for patients.
- Purpose/outcome – What was the purpose/end product of the activity?
  The nurse manager facilitates effective caring for patients by professional nurses through engagement, empowerment and support of professional nurses. The professional nurses provide effective caring for patients in PHC clinics.

## Setting

The setting of this study was the PHC clinics in Ekurhuleni, an area in the eastern part of Gauteng Province, South Africa. The PHC clinics provide comprehensive health care services such as maternal, child and reproductive health, human immunodeficiency virus, tuberculosis testing and treatment, screening and care for non-communicable diseases and treatment of common ailments through the DHS (Department of Health 2010:2). The PHC services are provided mainly by professional nurses, supported by doctors from the Gauteng Department of Health, as well as by doctors employed by City of Ekurhuleni municipality on a sessional basis of 2 h per day. Professional nurses are the largest category of health workers in the PHC clinics, with a limited number or no availability of other categories of staff such as enrolled nurses, enrolled nursing assistants, community health workers and pharmacist assistants in some PHC clinics. Majority of the PHC clinics only open 8 h a day during weekdays, staffed by 10 (*n* = 10) to 20 (*n* = 20) different staff categories, whilst a few also open on Saturdays for half a day. As revealed by the findings in Phase 1 of the main study, these PHC clinics are faced with challenges such as shortage of medicines, staff and functional medical equipment.

## Trustworthiness

Measures to ensure trustworthiness were ensured throughout the research study by using Guba’s model (Krefting 1991:215). Credibility was ensured through immersion in the field and making use of various strategies. The researcher ensured transferability by providing a rich description of the findings of Phase 1 in such a manner that another person can compare it to the findings of other studies (Holloway & Wheeler 2010:560; Houser 2015:238). A dense description of the research methodology supported by literature review was provided to ensure dependability. The quality of the developed strategies was enhanced by data triangulation and by synthesising (Anney 2014:277) the findings of Phase 1 with literature. To ensure confirmability (Polit & Beck 2017:560) the entire research process was supervised by the three study supervisors.

## Discussion of results

Three strategies were developed. These were as follows: Strategy 1, Facilitating maintaining of the empowering experiences by professional nurses; Strategy 2, Facilitating addressing of the disempowering experiences by professional nurses; and Strategy 3, Facilitating addressing of the disempowering PHC clinic systems. Table 1 outlines the developed strategies.

**TABLE 1:** The developed strategies.

Findings from previous study (Nesengani 2019:68)	Derived strategies
Empowering experiences in caring for patients: Effective caring for patients Appreciative behaviours by patients Constructive management practices	Strategy 1: Facilitating maintaining of the empowering experiences by professional nurses
Disempowering experiences in caring for patients: Negative experiences with colleagues Negative experiences with patients Negative experiences with management practices	Strategy 2: Facilitating addressing the disempowering experiences of professional nurses
Disempowering experiences with the PHC clinic systems Shortage of medicines, functional medical equipment, professional nurses High workloads Low salaries, no rewards and high professional nurses’ turnover Unavailable ambulances	Strategy 3: Facilitating addressing of the disempowering PHC clinic systems

Source: Nesengani, T.V., 2019, ‘Strategies for primary health care professional nurses to facilitate effective caring for patients in the primary health care clinics in Gauteng Province, South Africa’, PhD thesis, University of Johannesburg, viewed from http://hdl.handle.net/10210/412914.

PHC, primary health care.

The strategies will be discussed as follows:

### Strategy 1: Facilitating maintaining of the empowering experiences by professional nurses

This strategy focuses on maintaining empowering experiences such as effective caring for patients, effective communication and teamwork between professional nurses, as well as constructive management practices.

According to Andersson et al. (2015:1), effective caring is the essence of nursing and the central, dominant and unifying feature of nursing, which is the phenomenon related to restoring and maintaining patients’ health. Everett and Wright (2014:150) describe caring as person-centred, and very much at the forefront of contemporary policies; with its principles being concerned with the right of individuals to have their values and beliefs respected. The nursing profession further requires nurses to show caring behaviours that include comfort, compassion, concern, empathy, and love. Caring is the moral human trait, ethic or ideal at the heart of nursing (Delves-Yates 2015:173; Hawke-Eder 2017:23).

According to Makely, Badasch and Chesebro (2014:364) and Grover, Porter and Morphet (2017:92), effective communication and teamwork facilitates effective caring for patients. Effective communication skills enable professional nurses in this study to render effective caring through collaboration with one another (White, Cornish & Kerr 2017:196). According to Sallee (2014:327) and Elcock and Shapcott (2015:212), effective communication is a possible factor that enables the development of trust, honesty, integrity and dependability to one another, and the delivery of quality patient care is dependent on effective and efficient communication. Based on this, the value of effective communication and the need for trust in the healthcare setting is not only limited to the nurse-patient relationship, but rather pervades all working relationships. Makely et al. (2014:364) indicate that effective communication is more than just exchanging information and involves understanding the intentions behind the information.

The centrality of teamwork is to improve patient care planning. Working as a team enables PHC professional nurses in this study to overcome challenges in the workplace, which is also instrumental in boosting their morale. According to Hornby (2015:1552), teamwork is the act of working well together as a team. Sherwood (2014:399) elaborates that teamwork and collaboration are core competencies for the nursing practice, which enables nurses to function effectively as members of teams. Sherwood (2014:399) further indicates that teamwork manifests when two or more people interact interdependently with a common purpose, working towards measurable goals that maintain stability whilst encouraging honest discussion and problem-solving.

In this study, facilitating maintaining of empowering experiences such as constructive management practices brought about satisfaction amongst professional nurses. This further enhanced the working relationships between them and their management. Facilitating maintaining of empowering experiences through the promotion of the interpersonal empowering experiences was also to make the work relationships between professional nurses and the nurse managers successful. According to White et al. (2017:196), building of a functional working relationship is characterised by sharing a vision and a philosophy that enables people to respect each other. Building functional working relationships mainly allows people to value each other’s inputs and ideas. Employees feel valued and respected when they are heard by management. Therefore, healthy working relationships make the work more enjoyable (White et al. 2017:196).

### Strategy 2: Facilitating addressing the disempowering experiences of professional nurses

This strategy focuses on addressing the disempowering experiences identified as absenteeism, discrimination and negative attitudes, uncaring behaviours by professional nurses, as well as language barriers between professional nurses and patients. Hornby (2015:5) defines absenteeism as being frequently away from work or school, especially without good reason. Haskins et al. (2014:37), indicate that traditionally, absenteeism is an indicator of poor individual performance, breach of an implicit contract between employer and employee, as well as management problem. However, another identified factor attributed to high absenteeism in the workplace include poor morale, which is characterised by unhappy employees, reduced productivity and poor nursing care. In facilitating the addressing of the issue of absenteeism, professional nurses are encouraged to follow and respect the institution’s policies on absenteeism, which includes knowledge of processes and steps the employer can take against the employee in terms of staff absenteeism. They are also expected to discuss their intentions of going on leave with their managers a few weeks before the leave starts, as stipulated in the institution’s policies.

The need to address the scourge of discrimination and negative attitudes in this study was to stop exclusion or rejection based on the negative judgement towards patients as a result of their nationalities. Trenoweth and Allymamod (2015:225) indicate that knowledge on issues related to unfair discrimination and negative attitudes brings understanding amongst professional nurses that when they come into contact with someone who is disadvantaged because of something beyond that person’s control, they should make a conscious effort not to discriminate, but go out of their way to help the person any way they can. According to Sallee (2014:326), attitude involves a predisposition or tendency to respond in one way or another. The attitude that often accompanies a verbal interaction, can be positive or negative, and is described to be much more meaningful than the actual words spoken. Medical Human Rights Network (2013:1) mentions that refugee women experience specific abuses such as active discrimination and delayed or denied care. Stellenberg (2013:160) indicates that during clinical practice, nurses caring for patients must ensure that the care given is completely free of any form of unfair discrimination. According to Naidoo (2014:426), professional ethical standards do not tolerate acts of unfair discrimination directed towards patients. It is therefore recommended that care should be taken to deal fairly and equitably with all patients, regardless of ethnic origin, religion or other issues, so that no individual is treated less favourably; in order to deal with allegations of a lack of respect or unfair discrimination. Santana et al. (2017:432) mention that patient-centred care respects individual patient beliefs and values, promotes dignity and anti-discriminatory care for patients. In facilitating the strategy in this study, professional nurses are expected to show respect for diversity, embrace it by being tolerant and accommodative to all patients that they came into contact with, in order to foster a culture of putting patients first.

Stenhouse et al. (2016:12) indicate that the issues regarding the lack of quality care and uncaring behaviours stand in stark contrast to the popular stereotype of the caring and selfless, healing profession with a vocation epitomised by Nightingale and her peers. According to Knight (2012:3), in effective caring for patients, professional nurses engage in therapeutic use of self to enhance the working alliance that foster growth and improve health. Knight (2012:3) further highlights that in the nursing profession, therapeutic use of self describes how the nurse maintains a specific relationship with a patient and accepts responsibility for the actions in improving patients’ health. In this study, the professional nurses use themselves as therapeutic agents in effective caring for patients, which is exhibited by their expressions that they accept responsibility in their professional role.

According to Bowen (2015:2), those patients facing language barriers also face increased risks of medication errors, adverse events and complications, whilst the rights of limited English proficient patients to informed consent and confidentiality are often not protected. Wallmach (2013:393) highlights that when a patient understands what is being said about his or her caring, treatment and services, the patient is more likely to fulfil critical health caring responsibilities such as taking their medicines as prescribed. For communication to be effective, the information provided must be complete, accurate, and understood by the patient (Wallmach 2013:393). In addressing language barriers between professional nurses and patients in this study, professional nurses are to adopt a strategy of always functioning within the ethical framework of the nursing profession by integrating the values of respect professionalism and caring to build the PHC clinic’s climate that fosters effective caring for patients.

### Strategy 3: Facilitating addressing of the disempowering primary health care clinic systems

This strategy entails addressing the disempowering PHC clinic systems, identified as overcrowding of PHC clinics, which results in patients waiting for long to receive health care services, shortage of professional nurses; shortage of medicines, functional medical equipment and other essential resources. Facilitating addressing of the long queues of patients in the PHC clinics was regarded as an important issue as long queues led to long waiting times, which was perceived as a barrier to obtaining services by patients in Phase 1 of this study. Oche and Adamu (2013:588) indicate that the amount of time the patients wait to be seen is an important indicator of the quality of services, and is therefore, one factor which affects the utilisation of the services. Therefore, in facilitating the strategy, professional nurses engage themselves in the reduction of long queues and long waiting times for patients through queue management systems and by monitoring the patient waiting times. This is one of government’s requirements for effective caring for patients in healthcare facilities.

The shortage of professional nurses, which resulted in lack of capacity to render effective caring for patients in the PHC clinics in this study, placed an additional burden on the available staff. The nurse manager guided and encouraged professional nurses to raise their concerns regarding shortage of professional nurses with management in their staff meetings, which would help resolve the burden of the high workload they had to face on a daily basis. According to Igumbor et al. (2016:1), the majority of clinics experience a shortage of nursing staff, which is in line with the dire shortage of nurses in the public healthcare facilities in the country. Somahela, Yako and Khumalo (2015:178) highlight that clinics with low staff complement and higher workloads based on patient headcount, spend less time on patient consultations.

Shortage of medicines, functional medical equipment and other essential resources served as a barrier to render effective caring for patients by professional nurses. The shortage of medicines made it difficult for professional nurses to prescribe the treatment, right amount and right doses of treatment for patients. Bateman (2013:600) mentions that shortage of medicines results in some patients being turned away without any medicine at all, whilst others are told to buy over the counter. In some instances, disruptions in taking medicines increase the chances of patients’ infections becoming resistant to the drugs and failing to work. In addressing the shortage of medicines, lack of functional medical equipment and other resources in PHC clinics, professional nurses were guided to motivate their management to purchase adequate essential medicines and medical equipment, to enable effective caring for patients. The professional nurses were also empowered to motivate for the allocation of an adequate budget for fixing and servicing of the available medical equipment. In facilitating this strategy, timeous reporting by professional nurses of those medical equipment that are out of order, as well as those medicines that were out of stock also became crucial.

## Limitations of the study

The limitation for this study is that only the researcher and the study supervisors were involved in developing the strategies. The contributions from the professional nurses involved in caring for patients in PHC clinics would have enabled development of more strategies.

## Recommendations

Based on the findings of the study where shortcomings on caring for patients were identified, it is recommended that caring be considered as a major concept on which to build improvement. It is recommended that the strategies be regarded as a fundamental value to improve the culture of caring and to change the culture of how the professional nurses perform their work. This will ensure that respect, dignity, and better experiences for both professional nurses and patients are embedded in the nursing settings. To facilitate effective caring for patients by professional nurses in public PHC clinics in Ekurhuleni, it will be beneficial to implement and maintain the developed strategies to assist in resolving the identified problems. Facilitating maintaining of the empowering experiences by professional nurses will ensure good interpersonal relationships and effective communication. It will also be beneficial to implement the strategies that focus on facilitating addressing the disempowering experiences of professional nurses as this will enable them to address issues such as absenteeism, unfair discrimination, negative attitudes and uncaring behaviours. This will help them to treat patients fairly and with dignity. It will also be beneficial to implement the strategy that focus on facilitating addressing of the disempowering PHC clinic systems in order to resolve the issue of the shortage of professional nurses, medicines, functional medical equipment and other essential resources. Because of the limited research studies that focus on developing strategies to facilitate professional nurses to render effective caring for patients in PHC clinics, future studies that will focus on this aspect are recommended in order to add to the framework that guides nursing practice.

## Conclusion

This article reports on developing of strategies to facilitate effective caring for patients by professional nurses in PHC clinics. The themes derived from the findings of Phase 1 of the study and literature regarding facilitating 1) maintaining of the empowering experiences by professional nurses, 2) addressing the disempowering experiences of professional nurses as well as 3) addressing of the disempowering PHC clinic systems were the guiding principles in developing the strategies. The developed strategies will guide and assist professional nurses in PHC clinics in utilising effective caring behaviours displayed as kindness, empathy, respect and helpfulness in caring for patients within an ethical, reflective and knowing framework behaviours. The developed strategies will also enable professional nurses to maintain the empowering experiences and address their disempowering experiences in this environment.
